# Host immune responses to the itch mite, *Sarcoptes scabiei,* in humans

**DOI:** 10.1186/s13071-017-2320-4

**Published:** 2017-08-10

**Authors:** Sajad A. Bhat, Kate E. Mounsey, Xiaosong Liu, Shelley F. Walton

**Affiliations:** Inflammation & Healing Research Cluster, School of Health and Sport Sciences, Faculty of Science, Health, Education and Engineering, University of the Sunshine Coast, Locked Bag 4, Maroochydore DC, QLD 4558 Australia

**Keywords:** *Sarcoptes scabiei*, Scabies, Crusted scabies, Immune responses, Cytokines

## Abstract

Scabies is a parasitic disease due to infestation of skin by the burrowing mite *Sarcoptes scabiei.* Scabies is a major public health problem and endemic in resource poor communities worldwide affecting over 100 million people. Associated bacterial infections cause substantial morbidity, and in severe cases can lead to renal and cardiac diseases. Mite infestation of the skin causes localised cutaneous inflammation, pruritus, skin lesions, and allergic and inflammatory responses are mounted by the host against the mite and its products. Our current understanding of the immune and inflammatory responses associated with the clinical manifestations in scabies is far outweighed by the significant global impact of the disease. This review aims to provide a better understanding of human immune responses to *S. scabiei* in ordinary and crusted scabies phenotypes.

## Background

Scabies is an infestation of the skin caused by the burrowing ectoparasitic mite called *Sarcoptes scabiei* variety *hominis* (Greek word ‘sarx’ means flesh; ‘koptein’ means to smite or to cut and the Latin word ‘scabere’ means to scratch) [1]. It was reported in 2010 that about 100 million of the global population is infected with scabies [2] and prevalence in different regions ranged from 0.2 to 71.4% [3]. Scabies has been found to be more prevalent in developing countries and has a high impact on the health and social life of indigenous populations in developed countries [2]. In particular, countries of the Pacific and Latin American regions have a high burden of scabies and prevalence is substantially higher in children than in adolescents and adults [2, 3]. The global burden of scabies is reflected by the disability-adjusted life years (DALYs), a measure of health loss due to a disease or injury. One of the leading causes of skin-related DALYs in 2010 was scabies, with around 1.5 million DALYs attributable to scabies alone [4]. In addition to this direct burden, scabies is also linked to secondary complications such as rheumatic heart disease (RHD) and acute post-streptococcal glomerulonephritis (APSGN) [5]. These secondary complications if left untreated can lead to the development of serious downstream systemic and life-threatening conditions [6].

People with scabies suffer from intense itching mediated through allergic and inflammatory reactions mounted by the host against the mite and its products. A wide range of clinical features, from mild to severely destructive, occurs in scabies but despite the significant worldwide impact of the disease, the immune and inflammatory responses associated with the different clinical manifestations remain poorly characterized. This review focuses on the recent data which expands our knowledge of cellular and molecular mechanisms in immune responses to *S. scabiei* in Ordinary scabies (OS) and Crusted scabies (CS) in humans. In addition, the current understanding of scabies immunity will be compared and contrasted to responses in related parasitic infections and infestations.

### Clinical manifestations of scabies

Although a range of clinical presentations are apparent in scabies, for the purpose of this review we consider the two most commonly reported manifestations: OS (also known as classical or typical scabies) and CS (also known as Norwegian scabies, or scabies crustosa).

Ordinary scabies is the common form of scabies with a mite burden estimated to be less than 15 mites per person [7]. The main clinical signs include burrows, erythematous papules, and an allergic type skin reaction with intense, generalised pruritus. Occasionally, patients are asymptomatic [8]. Onset of the symptoms in a host with no previous infestation is delayed and occurs at 4 to 6 weeks’ post-infestation [9]. The primary papules may develop into secondary scabies lesions: excoriations and eczematisations. Patients usually show primary and secondary lesions existing together at the same time. Due to severe itching patients scratch the skin, opening up the lesion and making them susceptible to secondary bacterial infection.

Crusted scabies is relatively rare and an extreme manifestation with thousands of mites present which are same variant as those causing OS [10]. Due to the high number of mites present, CS is highly contagious as evidenced by nosocomial outbreaks of OS from index cases of CS [11]. Clinically, CS is a hyperkeratotic skin disease with thick and scaly crusts containing large numbers of mites. In CS patients, the infectivity persists for longer because of the difficulty in eradicating mites from heavily crusted skin. Mite reinfestation frequently occurs in the same individual and it is extremely debilitating and can cause permanent skin disfiguration. Crusted scabies patients may show deep fissuring of the crusts with pathogenic microbes gaining entry through these skin breaches and leading to serious secondary infections, frequently with the typical skin pathogens *Staphylococcus aureus* and *Streptococcus pyogenes*. Generalized lymphadenopathy due to secondary sepsis is common carrying high mortality rate if left untreated [9, 12, 13].

It is generally believed that immunosuppression and immunomodulation might be predisposing factors associated with CS. Crusted scabies has been shown in immunocompromised patients such as those with human immunodeficiency virus (HIV) infection [12], human T-lymphocytic virus 1 (HTLV-1) infection [14, 15] and in patients undergoing organ transplantation [16]. In addition, CS has been diagnosed in individuals with leprosy [14] and developmental disability, including Down’s syndrome, although the specific mechanisms linking these immune defects to crusted scabies have not yet been explored. Importantly, CS has also been detected in patients with no recognised immunodeficiency as evidenced in Aboriginal Australians [14, 17]. From these reports, it appears that the susceptibility of this cohort to CS may be due to a specific immune deficit, the nature of which is yet to be defined.

### Immune response in scabies

In animals, *S. scabiei* infestation (sarcoptic mange) results in inflammatory and adaptive immune responses relatively late in the infection (4–6 weeks after initial contact with mite), in contrast to related psoroptic mange where inflammatory responses are seen almost immediately after mite infestation. Given the parasite’s long co-evolution with its hosts, it is believed scabies mites have developed the capability of modulating various aspects of the host immune responses resulting in the delayed onset of symptoms [18, 19]. The rash and itch associated with scabies shows features of both type I (immediate) and type IV (delayed) hypersensitivity reactions. The initial inflammatory response as reviewed by Walton et al. [20] towards the mite and its products consists of Langerhans cells (LCs) and eosinophils with smaller number of monocytes, macrophages and mast cells.

### Innate immune responses

#### Complement system

The complement system is an essential and a far-reaching component of innate immunity and is the first line of defence against invading pathogens. It consists of almost 40 plasma and membrane associated proteins and together this complex network represents one of the major effector mechanisms of the innate immune system [21]. Complement proteins have been documented in host defence against blood-feeding ticks [22] and also in immune response to other pathogens [23]. Studies analyzing skin biopsies and circulating serum from scabies patients have revealed presence of complement components C3 and C4 [14, 24] suggesting both local and systemic sources of complement during infection. Complement fragments C3a and C4a act on specific receptors causing local inflammatory responses. In addition, C3a and C5a can activate mast cells to release mediators such as histamine and tumour necrosis factor alpha (TNF-α) that contribute to the inflammatory response [25]. The observation of these components in skin biopsies of CS patients [14] indicate an activated complement system which may be participating in the early inflammatory responses in scabies. Somewhat counterintuitively, low circulating C3, C4, or both have been reported in CS patients [14], suggesting some potential defect with complement function in CS, or possibly due to massive overload of mites and bacteria the system is unable to maintain production. Furthermore, there is evidence of scabies mite inactivated protease paralogues (SMIPPs) and serpins (SMSs) inhibiting complement activation and promoting bacterial growth in vitro, presumably protecting mites from complement mediated destruction [26, 27]. As suggested [28], production of such inhibitory molecules might be a way to evade host defence and also by promoting bacterial growth might provide further mechanisms contributing in disease pathogenesis. While these mechanisms still need to be defined in vivo, a recent study in a porcine model demonstrated the influence of scabies infestation on skin microbiota, with the microbial population changing from commensal to more pathogenic staphylococcal species [29]. Such studies are beginning to provide biological insights into the close association between scabies and bacterial skin infection.

#### Innate immune cells

The various innate effector cells detected in response to *S. scabiei* mites in OS and CS include eosinophils, mast cells, basophils, neutrophils, dendritic cells (DCs) and macrophages (Table 1). Eosinophils are produced in high numbers in allergic inflammation and helminth infections, and tissue eosinophilia is often found at inflammatory sites associated with these diseases [30]. Histological examination of 25 skin biopsies of scabies infection has shown the presence of dermal eosinophils in 22 patients with 68% of these showing numerous eosinophils and 20% of cases showing few eosinophils [31]. In CS, skin biopsy sections from two patients have shown large numbers of eosinophils in the dermis [24] and 58% of a cohort of CS patients were reported to have peripheral eosinophilia [14]. In *Psoroptes ovis* infested sheep and cattle, lesional histology studies also show an eosinophil dominated immunoinflammatory infiltrate [32, 33]. In addition, eosinophil infiltrations have been detected in the skin dermis of red foxes infested with *S. scabiei* [34]. This eosinophil detection is consistent with the high expression of T helper (Th) 2 representative cytokines interleukin (IL) 4, IL-5 and IL-13 in CS [35]. Eosinophils have been shown to express Th2 specific cytokines. IL-5 is involved in the attraction, activation and maturation of eosinophils and its production may be an autonomous mechanism for promoting the recruitment and survival of these granulocytes [30, 36]. The presence of eosinophils in CS and their ability to express Th2 profile cytokines [37] suggests that these granulocytes may themselves modulate or sustain the local Th2 inflammatory responses [38, 39] in scabies. Eosinophils may also regulate Th1 inflammatory response. Eosinophils have been shown to produce IL-12 and interferon gamma (IFN-γ) [40], and express several Toll-like receptors (e.g. Toll-like receptor 7) [41] which are part of innate immunity and responsible for Th1 biased responses. Furthermore, it is also suggested that eosinophil expression of IL-10 and transforming growth factor beta (TGF-β) may suppress local inflammatory responses by modulating the activities and development of regulatory T cells (Tregs). Alternatively, cytokine IL-2 is highly important in the development and survival of Treg cells [42] and eosinophil expression of IL-2 can result in the expansion of these T lymphocytes. In addition, eosinophil production of IL-10 and TGF-β [40, 43] may alter the local character of the Th2/Th1 responses by preventing the differentiation of naïve T lymphocytes to either the Th1 or Th2 phenotype [39]. By producing indoleamine 2, 3, −dioxygenase eosinophils may also drive Th1/Th2 imbalance [39]. Eosinophils are key players in defence against helminthic parasites but also contribute to tissue dysfunction and damage in allergic disease. However, the function and relative importance of eosinophils in the immune and inflammatory responses of both ordinary and crusted scabies is still undetermined.

**Table 1:** Immune response in scabies

	Ordinary scabies (OS)	Crusted scabies (CS)
Skin cellular responses	Mostly CD4^+^ T cells, eosinophils and macrophages [24]	Mostly CD8^+^ T cells, increased γδ^+^ T cells, eosinophils and few macrophages [14, 24, 35, 88]
Blood cell responses	T and B cells and T-cell subsets within normal ranges	T and B cells and T-cell subsets within normal ranges. Increased γδ^+^ T cells, eosinophilia [24, 88]
Th1/Th2 responses	Th1 mediated with increased production of Th1 cytokines IFN-γ, IL-2 and TNF-α [35, 46, 51]. Increased production of IL-10 [51]	Th2 mediated with increased production of Th2 cytokines IL-4, IL-5 and IL-13 [14, 35, 46]. Increased production of Th17 cytokines IL-17, IL −23 [46, 88]. Decreased production of IL-10 [24, 35]
Systemic Ig responses	Variable reports of elevated levels of total IgG, IgE, IgA and IgM. Increased levels of scabies-specific IgE, IgG and IgA [24, 35]	Increased levels of total IgG, IgG1, IgG3, IgG4, IgE and IgA. Elevated levels of scabies specific IgG4, IgE and IgA [24, 35]

Mast cells and basophils share morphological and functional similarities and are essential components in immunoglobulin (Ig) E mediated allergic diseases and the immune response to parasitic infections. Mast cells and basophils have been detected in skin lesions of scabies patients [44, 45], and in sheep with psoroptic mange [32]. In pigs, immunohistochemistry of skin lesions has revealed increased mast cells numbers in CS while their number remained steady over the course of infestation in OS [46]. A recent histological analysis of skin lesions of 86 red foxes with sarcoptic mange have shown numerous mast cells [47] and mast cells have also been detected in the dermis of free-living wombats with severe sarcoptic mange compared to normal wombats [48]. Upon activation, mast cells and basophils rapidly produce TNF-α, IL-6, Th2 cytokines IL-4, IL-5 and IL-13, which are the main molecules responsible for the allergic Th2-type inflammation [30, 49]. The mechanisms for the infiltration of mast cells and basophils into the blood and skin remains to be addressed to elucidate their role and importance in scabies inflammatory and allergic responses.

Macrophages, neutrophils, and DCs are immune effector cells involved in phagocytosis, antigen presentation and differentiation of T cells. These cells are associated with pro-inflammatory and allergic responses, parasitic infections and possibly humoral responses. IL-4, IL-13, TNF and IFN-γ play a role in alternative macrophage activation [50] and these cytokines have been reported in immune response to scabies [24, 35, 46, 51]. Macrophages, although in low numbers, have been detected in skin of patients with scabies [24] and cellular infiltrates of skin lesions in dogs infested with scabies mites [52, 53]. Low number of macrophages may be due to the production of immune modulating molecules secreted by the scabies mites. It has been suggested that early in the infestation mites inhibit the ability of macrophages to migrate to the site of inflammation allowing the mites to grow and establish [19].

Neutrophils are an essential part of the innate immune system. They drive the initiation of inflammation and are implicated as mediators of tissue-destructive events in various inflammatory diseases as previously reviewed [54, 55]. In a recent study, histological findings of skin lesions in 44 cases of bullous scabies revealed neutrophils as the predominant inflammatory cell infiltrates [56]. In another similar study, 25 skin biopsies obtained from scabies patients showed the presence of dermal neutrophils in 52% of cases [31]. Neutrophils have also been detected in inflammatory infiltrates in the skin of common wombats, sheep and red foxes infected with *S. scabiei* [34, 48, 57]. In an in vitro study using human whole blood, with *Staphylococcus aureus*, the recombinant *S. scabiei* mite protein SMSB4 was found to suppress bacterial killing by inhibiting opsonisation and phagocytosis by neutrophils [27].

Dendritic cells are among the first skin antigen presenting cells to come into contact with antigens, migrate to draining lymph nodes and process the antigens for presentation to effector T cells which results in T cell differentiation and activation. These cells are responsible for pathologies in infections, inflammatory disorders and have also been implicated in modulating the balance between immunity and peripheral tolerance [58, 59]. Histological analysis of the scabietic lesions of dogs have revealed infiltration of DCs in the skin epidermis [53] and DCs derived from human peripheral blood mononuclear cells (PBMCs) have been shown to secrete pro-inflammatory cytokines upon stimulation with scabies mite extract [60]. This engagement of DCs, neutrophils and macrophages in scabies warrants further investigations into their function, role and importance in immune and inflammatory responses in scabies mite infestations.

### Humoral immune responses

Scabies mite infestation is known to elicit robust antibody-mediated immune responses, especially in CS which is associated with extremely high levels of antigen specific IgG and IgE (Table 1). However, the timing of these responses, and their relative importance in establishing protective immunity remains poorly understood.

#### IgM

IgM is the first antibody to appear in response to antigen exposure and hence is traditionally considered the first line of the humoral immune response. In a recent study, ELISA analysis of serum in OS patients showed IgM antibodies that bound to scabies antigens in 74% of cases, although a canine antigen mite extract (*S. scabiei* var. *canis*) was used, and high cross-reactivity between antigens of scabies and house dust mites was shown which somewhat confounds the interpretation of this finding [61]. Nevertheless, these results suggest that IgM may be useful in detecting serum IgM to scabies antigens. IgM is the first antibody class to be produced and may allow earlier detection of scabies. However, given its low affinity for antigens and the cross-reactivity between house dust and scabies mite proteins the utility of IgM for serodiagnosis of scabies should be further investigated.

#### IgA

Secretory IgA is usually more abundant in mucosal regions than in serum and plays a critical role in immune function in the mucous membranes. In OS, it is not clear whether scabies-associated IgA secretion is increased or decreased as compared to non-infected individuals or CS, as studies have reported contradictory results [62–64]. Elevated levels of circulatory IgA were documented in 64% of study patients with CS [14]. In addition, Walton et al. [35] showed increased IgA binding to a recombinant scabies mite antigen in OS and CS patients compared to controls. In porcine studies, increased IgA serum levels in mange positive pigs was reported to whole mite antigen extract, with significant levels detected at week 10 in the infection and positively correlated with severity of infestation [65].

#### IgG

In animals, studies have demonstrated elevated serum levels of total IgG as compared to the controls in *S. scabiei* var. *canis* infested rabbits and dogs [62, 66–68]. Serum studies involving whole mite extracts of *S. scabiei* var. *suis* and Sars s 14.3 recombinant antigen demonstrated increased IgG, IgG1 and IgG2 responses in mange infected pigs from weeks 6–12 post-mite infestation [65]. *Sarcoptes scabiei* var. *ovis* primary infestation in sheep resulted in significant increases in the serum levels of specific IgG [69]. In the same study, secondary challenge in sheep induced a decreased IgG response in comparison to those observed during the primary infestation [69]. In goats with sarcoptic mange, analysis of antibody profiles has revealed a strong serum IgG response in primary infestation and repeated mite experimental challenges [70, 71]. In contrast, goats vaccinated with specific *S. scabiei* mite antigens showed high levels of scabies-specific IgG in the serum but this vaccination failed to provide protection against infestation despite the presence of elevated levels of IgG [70]. Further studies in dogs, where the IgG titres were inversely proportional to protection, also suggested that IgG antibodies conferred limited protection to sarcoptic mange in dogs [72], with similar conclusions in rabbits [62, 73]. These results also suggest that cell-mediated immune responses may be providing immune protection. In humans, mite infestations result in circulatory IgG responses in both OS and CS [24, 35, 74, 75] with CS patients showing stronger IgG responses compared to OS [74]. Increased serum levels of total IgG were reported in 56 of 58 of cases with CS [14]. On the other hand, only 27% of patients with OS showed circulatory IgG response directed at scabies mite antigens although *S. scabiei* var. *canis* mite extract was used which might limit sensitivity [61]. IgG subclass serology investigations have revealed elevated levels of total and antigen-specific IgG1, IgG3 and IgG4 in CS patients compared to non-infected controls [24, 35, 75]. Serum IgG4 levels are similarly elevated in chronic helminth and other parasite infections and also rise during allergy desensitization therapy, after repeated exposure to low doses of allergen [76]. The reason behind these elevated levels of total IgG and IgG isotypes especially in CS is unknown and may be due to high antigenic load imparted by mite hyper-infestation. Increases in total IgG could also result from concomitant bacterial infections [35]. Increased expression of IgG4 is likely due to the production of IL-4 and IL-13 in CS [14, 35] as these cytokines are known to drive antibody class switching and induce expression of IgG4 [77].

#### IgE

IgE is important in the host defence against to a variety of parasites and along with mast cells, basophils, and eosinophils, constitutes an essential element in allergic and parasitic inflammation. In humans, earlier studies have shown that scabies results in an increased production of circulating IgE antibodies but with highly divergent results [14, 63, 78, 79]. In recent studies, increased total IgE levels have been observed in OS patients [24, 51]. In a more recent study, ELISA analysis revealed only 2% of 91 cases with OS had circulating IgE antibodies that bound to *S. scabiei* var. *canis* antigens [61]. Conversely, OS patients from Australia had increased IgE antibodies specific to recombinant scabies antigens compared to naïve controls [35]. In another similar study, IgE binding to recombinant scabies mite antigen (Sar s 14) for OS was higher compared to controls, with a 100% diagnostic sensitivity and 94% specificity [80]. Furthermore, IgE binding to another scabies mite recombinant antigen was observed in patients with OS from Pakistan, and the ELISA used for detection showed high (over 90%) sensitivity and specificity (Naz S, personal communication). In comparison, in CS dramatic increases in total IgE levels have been consistently reported [24, 74] with one study showing 96% of 56 cases had elevated total IgE levels [14]. Immunoassay studies using plasma from subjects with CS showed increased specific IgE response to recombinant scabies mite molecules [35, 80]. Similar to IgG responses, as suggested by Roberts et al. [14] these results of increased IgE response in CS individuals are expected given the high amount of antigenic material/stimuli provided by the large number of mites. In the earlier studies reporting variable IgE changes, specific IgE responses to scabies mite antigens were not determined. In addition, altered serum IgE levels could be attributed to the different techniques and antigenic compositions of whole mite extracts used. For example, antigenic similarities exist between scabies and house dust mites, and IgE antibody cross-reactivity has been demonstrated [81, 82]. Hence studies using whole mite extracts may reflect a component of cross-reactive IgE binding. In contrast, the recent ELISA studies using various scabies mite-specific recombinant antigens show high specificity and sensitivity in IgE detection in both OS and CS phenotypes from different populations/demographics indicating their potential for serodiagnosis of scabies.

In animals, *S. scabiei* var. *canis* infestation in rabbits and dogs resulted in elevated serum levels of specific IgE [62, 66–68]. In sheep, primary *S. scabiei* var*. ovis* infestation resulted in significant increases in the serum levels of specific IgE and in secondary challenge a higher IgE response was observed than during the primary infestation [69]. In goats with sarcoptic mange, studies have demonstrated a strong IgE response in primary infestation and repeated mite experimental challenges [70]. IgE responses in pigs to *S. scabiei* mite infestation have not been explored as there is no porcine-specific IgE antibody commercially available.

While the above studies provide some insights into the humoral immune responses of scabies, a major limitation is the absence of robust prospective studies of human infestation. Such studies are difficult to perform due to the delayed appearance of symptoms and ethical considerations. Moreover, sensitivity is decreased when using non var. *hominis* mite antigen extracts, but obtaining sufficient amounts of scabies mites from human patients for research purposes to generate such extracts is logistically difficult, although recombinant antigens have been utilised [35, 65]. To further understand the humoral response in scabies it remains important to investigate the timing of onset of each Ig response; Ig profiles early in the infestation; after treatment and in reinfection; in both humans, and in suitable animal models; and the relationship between different antibody responses and their role in immunity. This information would be helpful for the development of improved diagnostic tools which would facilitate improved treatment and control of scabies at both the individual and community level.

In summary, in CS significantly higher levels of total and scabies-specific IgE and IgG antibody responses have been observed in comparison to OS where weaker and more varied responses are documented. From these studies, it also appears that differences may exist between the immune responses of humans and other animals to scabies, between primary and secondary infestations. These responses might also be affected by the sex of the host [71], type of infestation in humans (ordinary versus crusted) and the validity of the antigen used for diagnosis. Also, it seems that in some individuals/animals ineffective/dysregulated immune responses result in reduced acquired immunity to mite infestation [34, 71]. In addition, immunomodulation exerted by the mites appear to affect the immune response to infestation [18, 19, 83, 84]. This might explain why some animals/individuals fail to develop resistance to reinfection by *S. scabiei* and remained fully susceptible to recurrence of sarcoptic mange/scabies [34]. Also, this dysregulated/ineffective immune response early in the infestation in some individuals/animals might be playing a role in disease susceptibility especially to CS phenotype and the effects of these factors still need to be fully explored.

Elevated IgE and IgG responses have also been observed in parasitic infections such as in schistosomiasis and lymphatic filariasis [85–87] conferring protective immunity. However, the increased IgE and IgG scabies-specific antibody responses seen in CS seems not to be effective at clearing the scabies parasite, as shown by high rates of re-infestation [14] despite these increased antibody levels. It is suggested [14] that these increased serum levels of non-protective IgE and IgG in CS might be related to an inappropriate Th2 biased immune response but the reasons for this remain unknown.

### Cell-mediated immune responses

#### T cell infiltrates in *S. scabiei*-infested skin

T cell infiltrates to scabies mite infestation (Table 1) of the skin have been demonstrated in humans [24], pigs [88] and dogs [52, 53]. T cells are the main players in cell-mediated immune responses and cluster of differentiation (CD) 4^+^ T cells have been demonstrated as the most prevalent T lymphocytes in inflammatory skin lesions in OS (Table 1) in humans [89, 90] pigs and dogs [52, 88]. This corresponds to inflammatory cells in the skin lesions from patients with atopic dermatitis where a significantly greater number of infiltrating CD4^+^ lymphocytes compared with CD8^+^ subtypes is reported with CD4^+^ ⁄CD8^+^ ratios similar to peripheral blood levels [91].

In contrast, immunohistology and flow cytometry studies using biopsies from CS skin lesions of humans and pigs have revealed increased number of infiltrating CD8^+^ T cells (Table 1) compared with minimal or no CD4^+^ cells in the skin dermis [24, 88]. The number of T and B lymphocytes and T-cell subsets in the blood of CS patients have been reported within normal ranges [14, 24]. This presence of a greater number of CD8^+^ T cells in the skin than in the blood suggests a selective movement of CD8^+^ T cells. It is further hypothesised [24, 35] that these CD8^+^ T lymphocytes might be the cause of keratinocytes apoptosis leading to epidermal hyper-proliferation. This has also been observed in psoriasis patients with marked levels of CD8^+^ T cells in the skin epidermis and dermis [92]. The apoptotic keratinocytes may secrete cytokines which could exacerbate the inflammatory response by targeting resident skin cells causing further tissue damage. Therefore, these skin homing cytotoxic T cells may be responsible for the imbalanced inflammatory response and may contribute to the failure of the skin immune system to induce an effective response resulting in uncontrolled growth of the parasite. The precise role, importance and function of CD8^+^ T cells in the pathogenesis of CS needs to be investigated. Additionally, CD4^+^ T cells in the skin may be essential in the immune response to scabies conferring protection as it has been seen that acquired immunodeficiency syndrome (AIDS) patients often develop CS if infected with scabies mites [93, 94].

Recently our group reported that PBMC gamma delta (γδ)^+^ T cells increased in mange infected pigs relative to controls from as early as one week post mite infestation with these increases sustained throughout the infestation [88]. Similarly, strong PBMC proliferation of γδ^+^ T cells was reported in cattle infested with *P. ovis* [33]. These peripheral blood responses mirrored cutaneous responses, with skin cell infiltrates in the lesions of CS pigs showing significantly higher γδ^+^ T cell numbers than those with OS [88]. These elevated numbers of γδ^+^ T cells suggest their role in the disease pathogenesis as it has been demonstrated that IL-17 secretion by γδ^+^ T cells plays a critical role in the pathogenesis of psoriasis [95].

### Cytokine profiles in ordinary and crusted scabies

Cytokines, chemokines, and other mediators secreted by CD4^+^ (Th1, Th2, Th17 and Tregs) and CD8^+^ T cells along with other effector cells orchestrate the immune and inflammatory responses to scabies mite or its products (Table 2). These cells and their secreted molecules have been associated with specific immune responses and have been implicated in various inflammatory skin and infectious diseases.

**Table 2:** Effect of scabies mites or mite extracts on key cytokines and molecules from cultured cells in vitro and in vivo

Cell type(s)	Cytokines upregulated	Cytokines downregulated	Reference
Cultured cells treated with mite extracts			
Human skin equivalents (HSE)	CTACK, IL 1α, IL 1β, IL 1Rα, IL-6, IL-8, IL-23A, GM-CSF, M-CSF	Not reported	[18, 83]
Human dermal microvascular endothelial cells (HMVEC-D)	ICAM-1	IL-6, IL-8, VCAM-1	[114, 122]
Human keratinocytes, fibroblasts	IL-6, CTACK, TGF α, CXCL1, G-CSF	IL-8, GM-CSF	[84, 113]
Dendritic cells	TNF-α	IL-6, IL-8	[60]
In vivo studies: humans			
PBMCs	IL-10, IFN-γ, IL-6, IL-8, TNF-α, IL 1β, IL-4, IL-5, IL-13	IL-10	[35, 46, 57]
Serum	IL-10, TNF-α, IFN-γ	IL-6	[47]
Skin biopsies (crusted scabies)	IL-1β, TGF-β	IFN-γ, IL-10?	[23]
In vivo studies: other animals			
Porcine PBMCs	IL-17, IFN-γ	Not reported	[88]
Spleen (from exposed/vaccinated mice)	G-CSF, IL-2, IL-13	ICAM-1, ICAM-2, L-selectin, M-CSF, TNF α, TGF β	[112]
Canine PBMCs	IL-4, IL-5, TGF-β	TNF-α	[123]
Pig skin biopsies	IL-13, IL-17, IL-23, IL-4, IL-2, TGF-β	Not reported	[46]

Scabies mite infestation skews the Th1/Th2 immune response [96]. It is suggested that the host immune response to OS is a Th1 cell-mediated protective response. Th1-biased immune responses are dominated by CD4^+^ and CD8^+^ T cells secreting the cytokines IFN-γ, TNF-α and IL-2 [97, 98]. Studies have shown strong IFN-γ and TNF-α proliferative responses in PBMCs to scabies mite antigens [35] and studies have shown the presence of these cytokines in the serum of OS patients [51].

Th2 cells secrete IL-4, IL-5 and IL-13, and mediate humoral immunity by upregulating antibody production to fight extracellular parasites. Th2 cells are also dominant effector cells in the pathogenesis of IgE-mediated hypersensitivity in asthma and other allergic inflammatory diseases. PBMCs isolated from CS patients secreted increased levels of Th2 cytokines IL-4, IL-5 and IL-13, and decreased secretion of the Th1 cytokine IFN-γ as compared to OS upon stimulation with scabies mite antigens [35], similar to those seen with Der p 1 and HDM allergy [99]. Transcriptional analysis of skin biopsies from pigs with CS and cattle susceptible to severe psoroptic mange similarly revealed increased expression of IL-4, IL-5 and IL-13 [33, 46]. IL-4 and IL-13 play important roles in class switching of B cells and induce co-expression of IgE and IgG4 [100, 101], and thus the presence of these cytokines in CS is in alignment with the extremely high levels of IgG4 and IgE observed [14]. This strong humoral response in severe CS is not surprising given the antigenic load imparted by the hyper-proliferation of scabies mite. However, this Th2 skewed response may be a cause of rather than a response to scabies mite infestation, as porcine studies have demonstrated upregulation of these cytokines relatively early in infestation before mite numbers reach extremely high levels [46].

IL-17 is a potent proinflammatory cytokine, commonly recognised to be secreted by Th17 cells but it is also secreted by other cell types such as γδ^+^ and CD8^+^ T cells. Th17 cell generation and IL-17 secretion is promoted through cytokine signalling, in particular by IL-6, TGF-β, IL-23 and IL-1β or IL-18 [102]. Studies have demonstrated increased TGF-β, IL-23 and IL-1β expression in immune response to scabies mite infestation [18, 46], indicating that there are immune signals available in the local skin environment which may foster the generation of IL-17 secreting T cells. Furthermore, our group has shown increased IL-17 and IL-23 production in T cells isolated from skin lesions of pigs with CS [46, 88] and high levels of IL-17 have also been documented in the skin lesions of cattle breeds susceptible to psoroptic mange but not in those breeds resistant to *Psoroptes* infestation [33]. IL-17 secretion further exacerbates the ongoing inflammatory responses by inducing expression of TNF-α, IL-1β and IL-6 in epithelial cells as well as keratinocytes and fibroblasts [103]. Th17 cells and the cytokine IL-17 play a critical role in the inflammatory pathology associated with skin diseases, such as psoriasis and atopic dermatitis [104, 105] and parasitic *Leishmania major* and *Schistosoma japonicum* infections [106, 107].

TGF-β and IL-10 secreted by Tregs suppress pathological inflammatory responses [108]. It is suggested that Tregs may play a role in the control or development of scabies [88]. In CS, it has been demonstrated that IL-10 secretion in both PBMCs and lesional skin is significantly reduced compared to OS and control cases [24, 35]. In agreement with this, Abd El-Aal et al. [51] reported a negative correlation between IL-10 secretion and severity of lesions in ordinary scabies. It has been postulated that the activity of Tregs and IL-10 secretion [47] in OS cases may contribute to inhibition of inflammatory and immune reactions to the parasite which may partially explain the 4–6 week incubation period in a primary infestation of *S. scabiei*. It has been demonstrated in PBMCs that scabies mite extract can induce IL-10 expression and by extrapolation, influence Tregs activity [109]. The delay in symptoms may also be in part due to the well-known ability of IL-10 in inhibiting synthesis of the proinflammatory cytokines TNF-α, IFN-γ an IL-2 [110, 111]. It is suggested that this reduced IL-10 expression may cause expansion of IL-17-secreting T cells resulting in a Treg/Th17 dysfunctional immune response. Such a hypothesis is supported by a recent murine study of mucocutaneous leishmaniosis, where blocking of IL-10R resulted in increased IL-17 responses and more severe skin pathology [106].

### Summary, recent developments and future directions

**Fig. 1:**
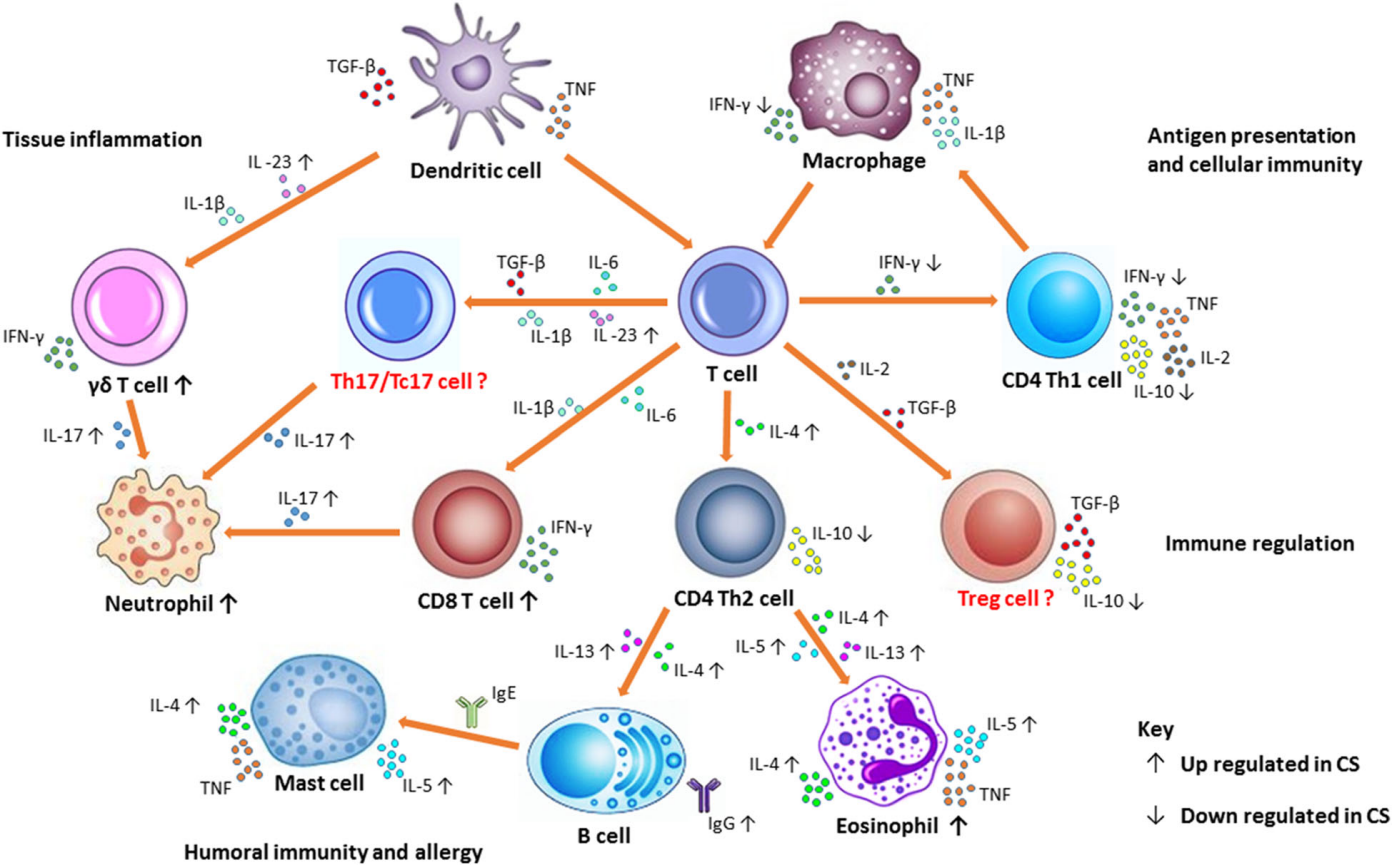
Current knowledge on immune mechanisms in scabies: The figure shows possible mechanisms of immune responses to scabies mite infestation. Keratinocytes, langerhans cells, and macrophages in the skin respond to mite antigens, secreting proinflammatory cytokines such as TNF-α, IFN-γ, TGF-β, IL-1β and IL-23. This leads to the differentiation and recruitment of CD8^+^ T and CD4^+^ Th1 and Th2 cells into the skin. Secreted cytokine milieus of IL-6, TGFβ and IL-23 promote the differentiation of Th17 or Tc17 cells and IL-17 production. IL-23 and IL-1β also have firmly established roles in promoting IL-17 production by γδ T cells, and their increased expression observed in CS may act in an amplification loop for IL-17 production, promoting inflammation and aggravating immune pathology. TGF-β and IL-2 induce Tregs. IL-10 and TGF-β production by Tregs may contribute to the delayed inflammatory response in scabies and suppress pathological inflammation in ordinary scabies, regulating innate and adaptive responses. Immune responses to ordinary scabies appear Th1 oriented as evidenced by strong IFN-γ secretion in response to mite antigens. Increased expression of the Th2 cytokines IL-4 and IL-3 in CS leads to immunoglobulin switching in B cells resulting in secretion of large amounts of IgE and IgG. IL-5 activates and promotes the maturation of eosinophils at the site of infestation, sustaining the local Th2 inflammatory responses. IgE through its high affinity receptor (FcεRI) activates mast cells. These cells produce inflammatory mediators such as TNF, histamine, leukotrienes, IL-4, IL-5 and IL-13, supporting their contribution to allergic inflammation in CS. Cells highlighted in “red” and with “?” are not yet defined in scabies

In summary, our current understanding indicates that the immune responses to scabies are complex, with distinct profiles between the different clinical manifestations (summarised in Fig. 1, Table 1). Crusted scabies shows a picture of increased CD8^+^ and γδ^+^ T cell infiltration [24, 88], increased production of IgE [14, 24, 35], elevated secretion of Th2 cytokines IL4, IL-5 and IL-13, decreased IL-10 production [35] and increased production of Th17 cytokines IL-17 and IL-23 [88] suggesting a mix of non-protective allergic Th2 and IL-17 responses contributing in disease pathogenesis. However, the underlying mechanisms of these elevated responses in CS is not yet known and the knowledge about these events is important in understanding the development and immunological progression in CS. Although CS is often associated with general immunosuppressive conditions such as HIV, HTLV-1 infections, or patients undergoing organ transplantation, some people with no recognised immunodeficiency still develop CS [14]. In addition, studies with pigs have shown that some pigs develop OS while others exhibit CS phenotype after infestation following inoculation with a similar number of mites [65, 88]. This indicates that these individuals/animals may have an immunity related genetic predisposition increasing susceptibility to CS. These genetic changes may not directly cause CS but may play a role in its development. Detailed gene expression studies would be beneficial for identifying such genetic changes, particularly if conducted early in infestation, prior to the development of high mite burdens and severe clinical pathology with confounding co-morbidities.

One of the characteristic features of a primary *S. scabiei* infestation is that clinical signs of cutaneous inflammation and pruritus do not appear until weeks after infestation. Studies show that *S. scabiei* may be inhibiting the early immune responses by downregulating the expression of proinflammatory mediators and cytokines [18, 60, 112–114]. However, these studies were mostly in vitro, utilising mite extracts and cultured cells or skin equivalents.

To gain valuable insights into the mechanisms behind the immune and inflammatory responses driving disease outcomes in scabies, it is highly imperative to look into the immune responses upstream of those seen at clinical presentation, with in vivo prospective studies. As access to patients with scabies is very limited and carrying out a study of infection in humans can be logistically and ethically a difficult process, thus animal models provide an alternative to investigate the immunopathological mechanisms of scabies development. In addition to studies undertaken with experimental infestation in dogs [52, 53] and rabbits [66], a model involving pigs has been recently utilised [115]. Pigs are a natural host to *S. scabiei* var. *suis* and develop clinical manifestations resembling both CS and OS, making this model preferable for comparative immunology studies [116].

This porcine model has already allowed more detailed study of scabies immunology than previously possible and importantly validated critical findings from human crusted scabies where interpretation was limited by small sample numbers. Further studies have also begun to shed some light into the effect of scabies mite infestation on the skin microbiota, and helping to unravel the complexities between *S. scabiei* and concomitant bacterial infection [117]. Recent genome sequencing [118, 119] and proteomic analysis [120, 121] of *S. scabiei* promises to provide further insights in understanding the biology of the mite, molecular basis of host specificity, host-parasite interactions, parasitic adaptations and immune evasion. The knowledge of host immune responses and genetic changes in scabies is essential and may aid in the development of novel therapeutics, diagnostic and disease control, as well as allow the early discrimination of ordinary scabies from the severe form of the disease.

## Conclusion

In conclusion, development of immunodiagnostics, vaccines, and immunotherapeutic represents a promising long term strategy to control scabies in affected communities globally. A comprehensive understanding of the immune events in the skin and peripheral blood occurring during scabies may provide multiple points at which immunological interventions may intersect the infection and target the responses away from pathology to immunity.

## Abbreviations

AIDS: Acquired Immunodeficiency Syndrome
C: Complement
CD: Cluster of differentiation
CS: Crusted scabies
CTACK: Cutaneous T-cell-attracting chemokine
CXCL: Chemokine (C-X-C motif) ligand
DC: Dendritic cell
ELISA: Enzyme-linked immunosorbent assay
GM-CSF: Granulocyte-macrophage colony-stimulating factor
ICAM: Intercellular adhesion molecule
IFN-γ: Interferon-γ
Ig: Immunoglobulin
IL-10: Interleukin-10
IL-12: Interleukin-12
IL-13: Interleukin-13
IL-18: Interleukin-18
IL-1β: Interleukin-1 beta
IL-2: Interleukin 2
IL-22: Interleukin-22
IL-23: Interleukin-23
IL-4: Interleukin-4
IL-5: Interleukin-5
IL-6: Interleukin-6
IL-8: Interleukin-8
M-CSF: Macrophage colony-stimulating factor
OS: Ordinary scabies
PBMCs: Peripheral blood mononuclear cells
SMIPPs: Scabies mite proteases
SMSs: Scabies mite serpins
TGF-β: Transforming growth factor-beta
Th: T helper
TNF: Tumour necrosis factor
Tregs: Regulatory T cells
Γδ: Gamma delta
